# The spleen mediates chronic sleep restriction-mediated enhancement of LPS-induced neuroinflammation, cognitive deficits, and anxiety-like behavior

**DOI:** 10.18632/aging.103659

**Published:** 2020-08-03

**Authors:** Dan Xu, Yujing Zhang, Bing Xie, Hua Yao, Yin Yuan, Shiying Yuan, Jiancheng Zhang

**Affiliations:** 1 Department of Critical Care Medicine, Union Hospital, Tongji Medical College, Huazhong University of Science and Technology, Wuhan 430022, China; 2 Institute of Anesthesia and Critical Care Medicine, Union Hospital, Tongji Medical College, Huazhong University of Science and Technology, Wuhan 430022, China

**Keywords:** chronic sleep restriction, microglia, neuroinflammation, sepsis, spleen

## Abstract

Chronic sleep restriction promotes neuroinflammation and cognitive deficits in neurodegenerative and neurobehavioral diseases. The spleens of mice exposed to chronic and repeated psychological stress serve as a reservoir of inflammatory myeloid cells that are released into the blood and brain following secondary acute stress. Here, we tested whether chronic and repeated short-term sleep restriction (CRSR) would exacerbate lipopolysaccharide (LPS)-induced neuroinflammation, cognitive deficits, and anxiety-like behavior in a spleen-dependent manner. LPS was administered to aged mice 14 days after exposure to CRSR consisting of three cycles of 7 days of sleep restriction with 7-day intervals in between. CRSR increased plasma proinflammatory cytokine levels, blood-brain barrier permeability, hippocampal proinflammatory cytokine levels, and transition of microglia to the M1 phenotype 24 h after LPS treatment. This in turn led to cognitive deficits and anxiety-like behavior. Interestingly, removal of the spleen 14 days prior to CRSR abrogated the enhancement of LPS-induced increases in systemic inflammation, neuroinflammation, cognitive deficits, and anxiety-like behavior. These data indicate that the spleen was essential for CRSR-induced exacerbation of LPS-induced brain damage.

## INTRODUCTION

Sleep is a physiological and behavioral process that plays an important role in metabolism, immune system homeostasis, and brain development and plasticity [1, 2]. Mood disorders, cognitive impairment, deficits in attention and sensorial perception, metabolic dysfunction, inflammation, and immune dysfunction are all increased in individuals experiencing sleep disturbances [2–8]. Chronic sleep disturbances are pervasive in modern society due to poor lifestyle habits, stressful working conditions, and aging [9]. Sleep and circadian disturbances are common in older adults due to their reduced ability to initiate and maintain sleep [10, 11]. Chronic sleep disturbances are associated with systemic inflammation as well as cellular and humoral immune system dysfunction [12]. Evidence also indicates that chronic sleep restriction can promote neuroinflammation, synapse loss, mood disorders, and cognitive impairment in neurodegenerative and neurobehavioral diseases [12–15]. LPS-induced systemic inflammation can disrupt the blood-brain barrier (BBB) and activate microglia and neuroinflammation, ultimately leading to cognitive deficits [16–18]. Evidence has shown that chronic insufficient sleep exacerbates febrile responses to lipopolysaccharide (LPS)-induced inflammation in mice [19]. However, the roles and mechanisms of chronic and repeated sleep restriction in LPS-induced systemic inflammation, neuroinflammation, cognitive deficits, and anxiety-like behaviors remain unclear.

Previous studies demonstrated that spleens from mice repeatedly exposed to psychological stress act as a reservoir of primed monocytes that were released into the blood following sympathetic activation by secondary acute stress injury and eventually reached the brain [20, 21]. Furthermore, splenic reservoirs of myeloid progenitor cells are the source of these circulating monocytes [20], and removal of the spleen prior to repeated psychological stress prevented monocyte trafficking to the blood and brain [21]. Additionally, myeloid cells that accumulate in the spleen can be redistributed to tumor tissues after chronic psychological stress, and splenectomy can prevent chronic stress-induced increases in myeloid cells in tumor tissues [22]. The spleen also mediates inflammation and distribution of blood natural killer cells, monocytes, and polymorphonuclear myeloid-derived suppressor cells after psychological stress [23]. These findings indicate that the spleen is an important link between chronic psychological stress and acute stress injury-induced systemic immuno-inflammatory responses.

In this study, we investigated whether chronic and repeated short-term sleep restriction (CRSR) could exacerbate systemic inflammation, hippocampal neuroinflammation, cognitive deficits, and anxiety-like behavior in an LPS-induced systemic inflammation mouse model. We also examined whether the spleen mediated LPS-induced effects in CRSR-exposed mice.

## RESULTS

### CRSR exacerbated LPS-induced cognitive deficits, anxiety-like behavior, and systemic inflammation

**Figure 1:**
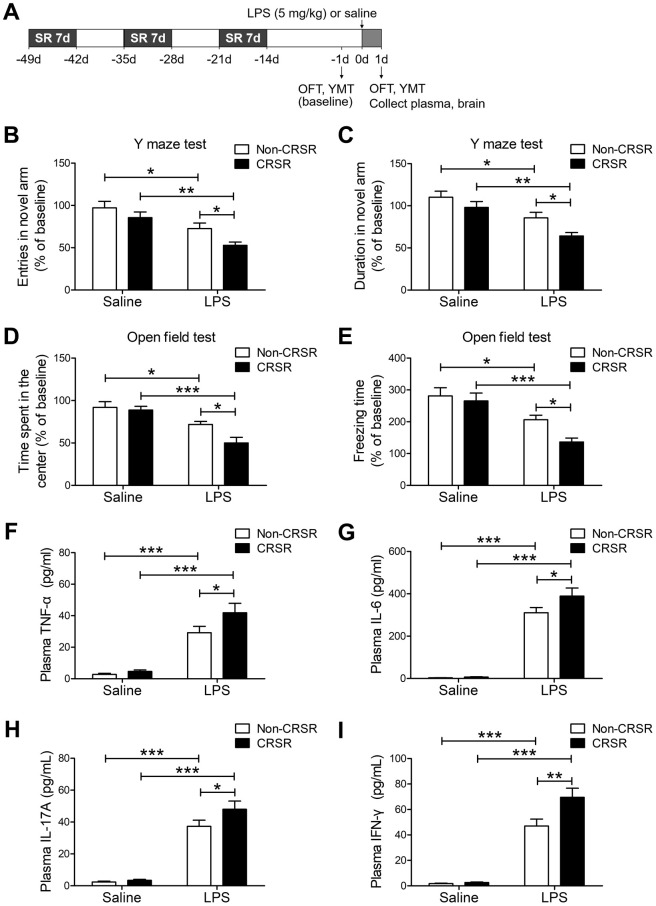
**Effects of chronic and repeated short-term sleep restriction (CRSR) on lipopolysaccharide (LPS)-induced cognitive deficits, anxiety-like behavior, and systemic inflammation**. (**A**) Experimental schematic. Adult mice were subjected to CRSR consisting of 3 repeated cycles of 7-day sleep restriction with an interval of 7 days. LPS (5 mg/kg) or 0.9% saline (5 mL/kg) was administrated intraperitoneally 14 days after the last cycle of sleep restriction. In the Y maze test (YMT), the number of entries (**B**) and time spent in the novel arm (**C**) were assessed in each group 1 day prior to LPS treatment as a baseline and 2 days after LPS treatment. LPS-induced decreases in the number of entries and time spent in the novel arm were exaggerated by CRSR. In the OFT, time spent in the center (**D**) and freezing time (**E**) were assessed in each group 1 day prior to LPS treatment as a baseline and 2 days after LPS treatment. LPS-induced decreases in time spent in the center and freezing time were exaggerated by CRSR. Plasma was collected 24 hours after LPS treatment for enzyme linked immunosorbent assay (ELISA) detection of TNF-α (**F**), IL-6 (**G**), IL-17A (**H**) and IFN-γ (**I**) in each group. LPS-induced increases in hippocampal TNF-α, IL-6, IL-17A, and IFN-γ levels were exaggerated by CRSR. Data represent means ± SEM, n = 6; *P < 0.05, **P < 0.01, ***P < 0.0001.

First, we studied the effects of CRSR on cognitive deficits and anxiety-like behavior in the LPS-induced systemic inflammation mouse model. The open field test (OFT) and Y maze test (YMT) can successfully detect anxiety-like behavior and working memory dysfunctions in mice [24]. Two-way ANOVA analysis of Y maze test data showed that CRSR decreased number of entries (CRSR: F_1,20_ = 6.206, P = 0.0216; LPS: F_1,20_ = 20.54, P = 0.0002; interaction (CRSR × LPS): F_1,20_ = 0.4226, P = 0.5230) and time spent in the novel arm (CRSR: F_1,20_ = 7.014, P = 0.0154; LPS: F_1,20_ = 21.21, P = 0.0002; interaction (CRSR × LPS): F_1,20_ = 0.5378, P = 0.4719) compared to the non-CRSR group in LPS-treated mice, but not in saline-treated mice (Figure 1B, 1C).

Two-way ANOVA analysis of OFT data showed that CRSR decreased time spent in the center (CRSR: F_1,20_ = 4.988, P = 0.0371; LPS: F_1,20_ = 28.22, P < 0.0001; interaction (CRSR × LPS): F_1,20_ = 2.827, P = 0.1082) and freezing time (CRSR: F_1,20_ = 4.567, P = 0.0451; LPS: F_1,20_ = 25.77, P < 0.0001; interaction (CRSR × LPS): F_1,20_ = 1.840, P = 0.1901) compared to the non-CRSR group in LPS-treated mice, but not in saline-treated mice (Figure 1D, 1E).

After behavioral testing, levels of pro-inflammatory cytokines in the plasma were measured to elucidate the mechanisms by which CRSR enhanced LPS-induced cognitive deficits and anxiety-like behavior. CRSR increased TNF-α (CRSR: F_1,20_ = 3.898, P = 0.0623; LPS: F_1,20_ = 75.38, P < 0.0001; interaction (CRSR × LPS): F_1,20_ = 2.178, P = 0.1556), IL-6 (CRSR: F_1,20_ = 3.291, P = 0.0847; LPS: F_1,20_ = 232.7, P < 0.0001; interaction (CRSR × LPS): F_1,20_ = 2.734, P = 0.1138), IL-17A (CRSR: F_1,20_ = 3.291, P = 0.0847; LPS: F_1,20_ = 149.6, P < 0.0001; interaction (CRSR × LPS): F_1,20_ = 2.244, P = 0.1498), and IFN-γ (CRSR: F_1,20_ = 6.785, P = 0.0169; LPS: F_1,20_ = 156.3, P < 0.0001; interaction (CRSR × LPS): F_1,20_ = 5.836, P = 0.0254) protein expression compared to the non-CRSR group in LPS-treated mice, but not in saline-treated mice (Figure 1F–1I).

### CRSR exacerbated LPS-induced increase in BBB disruption and hippocampal proinflammatory cytokine levels

**Figure 2:**
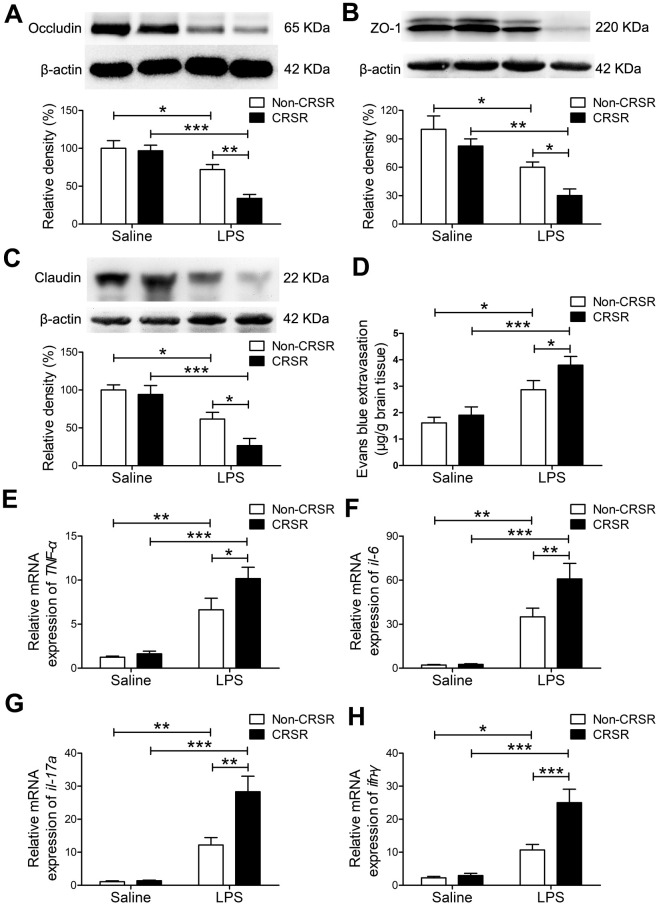
**Effects of chronic and repeated short-term sleep restriction (CRSR) on lipopolysaccharide (LPS)-induced blood-brain barrier (BBB) disruption and hippocampal inflammation 24 hours after LPS treatment.** Western blot analysis of the expression of the tight junction proteins occludin (**A**), zona occluden-1 (ZO-1; **B**) and claudin (**C**) in the brain. (**D**) Evans blue dye extravasation test. LPS-induced decreases in hippocampal tight junction protein levels and increase in Evans blue dye extravasation into the brain were exaggerated by CRSR. Quantitative real-time PCR (qRT-PCR) analysis of hippocampal gene expression of TNF-α (**E**), IL-6 (**F**), IL-17A (**G**), and IFN-γ (**H**). The LPS-induced increase in hippocampal expression of these genes was exaggerated by CRSR. Data represent means ± SEM, n = 6; *P < 0.05, **P < 0.01, ***P < 0.0001.

Next, we examined the effects of CRSR on BBB permeability and hippocampal proinflammatory cytokine levels after LPS treatment. Reduced BBB integrity is associated with decreased levels of the tight junction proteins occludin, ZO-1, and claudin [25]. CRSR decreased occludin (CRSR: F_1,20_ = 7.446, P = 0.0129; LPS: F_1,20_ = 36.38, P < 0.0001; interaction (CRSR × LPS): F_1,20_ = 5.317, P = 0.0320), ZO-1 (CRSR: F_1,20_ = 6.692, P = 0.0176; LPS: F_1,20_ = 25.11, P < 0.0001; interaction (CRSR × LPS): F_1,20_ = 0.4450, P = 0.5124), and claudin (CRSR: F_1,20_ = 4.668, P = 0.0430; LPS: F_1,20_ = 31.48, P < 0.0001; interaction (CRSR × LPS): F_1,20_ = 2.374, P = 0.1390) protein expression in the hippocampus compared to the non-CRSR group in LPS-treated mice, but not in saline-treated mice (Figure 2A–2C).

Evans blue is the most commonly used marker of blood-brain barrier integrity, and its extravasation is indicative of BBB breakdown [26, 27]. CRSR increased Evans blue extravasation into the brain (CRSR: F_1,20_ = 3.989, P = 0.0596; LPS: F1,20 = 26.58, P < 0.0001; interaction (CRSR × LPS): F1,20 = 1.095, P = 0.3079) compared to the non-CRSR group in LPS-treated mice, but not in saline-treated mice (Figure 2D).

Evidence indicates that increased BBB permeability can increase inflammatory cytokine infiltration of the brain in septic mice exposed to sleep fragmentation [28, 29]. CRSR increased TNF-α (CRSR: F1,20 = 4.388, P = 0.0491; LPS: F1,20 = 55.35, P < 0.0001; interaction (CRSR × LPS): F1,20 = 2.828, P = 0.1082), IL-6 (CRSR: F1,20 = 4.715, P = 0.0421; LPS: F1,20 = 56.85, P < 0.0001; interaction (CRSR × LPS): F1,20 = 4.400, P = 0.0488), IL-17A (CRSR: F1,20 = 9.860, P = 0.0052; LPS: F1,20 = 53.04, P < 0.0001; interaction (CRSR × LPS): F1,20 = 9.144, P = 0.0067), and IFN-γ (CRSR: F1,20 = 11.26, P = 0.0031; LPS: F1,20 = 46.44, P < 0.0001; interaction (CRSR × LPS): F_1,20_ = 9.244, P = 0.0065) mRNA levels in the hippocampus compared to the non-CRSR group in LPS-treated mice, but not in saline-treated mice (Figure 2E–2H).

### CRSR exacerbated LPS-induced transition of microglia to M1 phenotype

**Figure 3:**
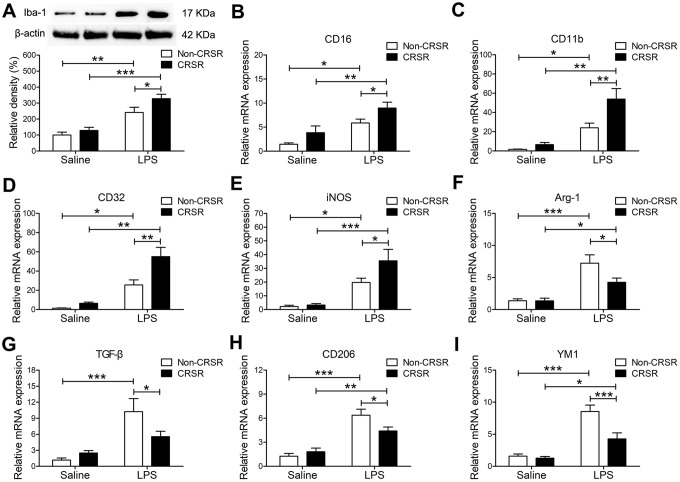
**Effects of chronic and repeated short-term sleep restriction (CRSR) on lipopolysaccharide (LPS)-induced transformation of microglia to M1 phenotype 24 hours after LPS treatment.** (**A**) Western blotting analysis of hippocampal ionized calcium-binding adapter molecule 1 (Iba1) expression in each group. The LPS-induced increase in hippocampal Iba1 expression was exaggerated by CRSR. Quantitative real-time PCR (qRT-PCR) analysis of hippocampal expression of the microglial M1 markers CD16 (**B**), CD11b (**C**), CD32 (**D**), and iNOS (**E**) and the M2 markers Arg-1 (**F**), TGF-β (**G**), CD206, (**H**) and YM1 (**I**). LPS-induced increases in microglial M1 markers and decreases in M2 markers in the hippocampus were exaggerated by CRSR. Data represent means ± SEM, n = 6; ^*^P < 0.05, ^**^P < 0.01, ^***^P < 0.0001.

LPS-induced systemic inflammation activates microglia and promotes their polarization toward the M1 phenotype [30]. The microglia marker ionized calcium-binding adapter molecule 1 (Iba1) is expressed at all microglia activation stages, and its expression increases as activation progresses [31]. CRSR increased hippocampal Iba1 expression (CRSR: F_1,20_ = 5.280, P = 0.0325; LPS: F_1,20_ = 46.12, P < 0.0001; interaction (CRSR × LPS): F_1,20_ = 1.303, P = 0.2672) compared to the non-CRSR group in LPS-treated mice, but not in saline-treated mice (Figure 3A). Moreover, CRSR increased levels of the microglial M1 markers CD16 (CRSR: F_1,20_ = 7.132, P = 0.0147; LPS: F_1,20_ = 21.41, P = 0.0002; interaction (CRSR × LPS): F_1,20_ = 0.1226, P = 0.7299), CD11b (CRSR: F_1,20_ = 8.205, P = 0.0096; LPS: F_1,20_ = 32.91, P < 0.0001; interaction (CRSR × LPS): F_1,20_ = 4.186, P = 0.0546), CD32 (CRSR: F_1,20_ = 9.851, P = 0.0052; LPS: F_1,20_ = 43.74, P < 0.0001; interaction (CRSR × LPS): F_1,20_ = 4.925, P = 0.0382), and iNOS (CRSR: F_1,20_ = 3.594, P = 0.0725; LPS: F_1,20_ = 31.05, P < 0.0001; interaction (CRSR × LPS): F_1,20_ = 2.712, P = 0.1152), but decreased levels of the microglial M2 markers Arg-1 (CRSR: F_1,20_ = 3.691, P = 0.0691; LPS: F_1,20_ = 31.54, P < 0.0001; interaction (CRSR × LPS): F_1,20_ = 3.609, P = 0.0720), TGF-β (CRSR: F_1,20_ = 1.524, P = 0.2313; LPS: F_1,20_ = 20.08, P = 0.0002; interaction (CRSR × LPS): F_1,20_ = 4.848, P = 0.0396), CD206 (CRSR: F_1,20_ = 1.736, P = 0.2026; LPS: F_1,20_ = 52.79, P < 0.0001; interaction (CRSR × LPS): F_1,20_ = 5.614, P = 0.0280), and YM1 (CRSR: F_1,20_ = 10.04, P = 0.0048; LPS: F_1,20_ = 47.37, P < 0.0001; interaction (CRSR × LPS): F_1,20_ = 7.482, P = 0.0127) compared to the non-CRSR group in LPS-treated mice, but not in saline-treated mice (Figure 3B–3I).

### Splenectomy blocked the enhancing effects of CRSR on LPS-induced cognitive deficits, anxiety-like behavior, and systemic inflammation

**Figure 4:**
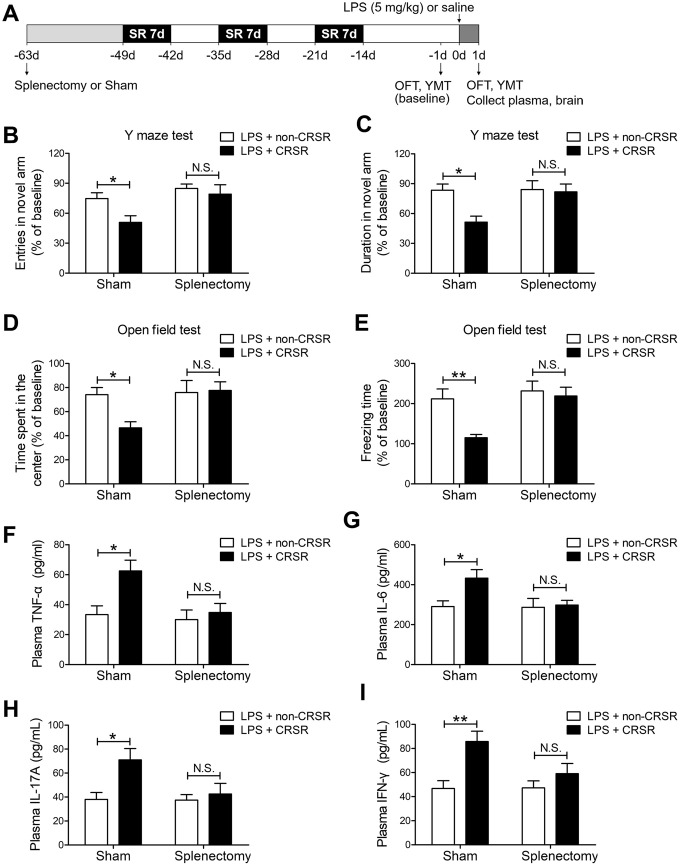
**The spleen mediates the enhancing effects of chronic and repeated short-term sleep restriction (CRSR) on LPS-induced cognitive deficits, anxiety-like behavior, and systemic inflammation.** (**A**) Experimental schematic. Splenectomy or sham-splenectomy was performed 14 days prior to the first cycle of sleep restriction. In the Y maze test (YMT), the number of entries (**B**) and time spent in the novel arm (**C**) were assessed in each group 1 day prior to LPS treatment as a baseline and 2 days after LPS treatment. Splenectomy blocked CRSR-mediated exacerbation of LPS-induced decreases in the number of entries into the novel arm and time spent in the novel arm. In the OFT, time spent in the center (**D**) and freezing time (**E**) were also assessed in each group 1 day prior to LPS treatment as a baseline and 2 days after LPS treatment. Splenectomy blocked CRSR-mediated exacerbation of LPS-induced decreases in time spent in the center and freezing time. Plasma was collected 24 hours after LPS treatment for enzyme linked immunosorbent assay (ELISA) detection of TNF-α (**F**), IL-6 (**G**), IL-17A (**H**), and IFN-γ (**I**). Splenectomy blocked CRSR-mediated exacerbation of LPS-induced increases in plasma TNF-α, IL-6, IL-17A, and IFN-γ levels. Data represent means ± SEM, n = 6; ^*^P < 0.05, ^**^P < 0.01. N.S., not significant.

To determine the role of spleen in CRSR-mediated exacerbation of LPS-induced injury, splenectomy was performed 14 days prior to CRSR. Two-way ANOVA analysis of Y maze test results revealed that splenectomy blocked CRSR-mediated exacerbation of the LPS-induced decrease in number of entries into the novel arm (LPS + CRSR: F_1,20_ = 4.670, P = 0.0430; Splenectomy: F_1,20_ = 7.866, P = 0.0109; interaction ((LPS + CRSR) × Splenectomy): F_1,20_ = 1.754, P = 0.2004) and time spent in the novel arm (LPS + CRSR: F_1,20_ = 5.583, P = 0.0284; Splenectomy: F_1,20_ = 4.554, P = 0.0454; interaction ((LPS + CRSR) × Splenectomy): F_1,20_ = 4.177, P = 0.0544) (Figure 4B, 4C).

Two-way ANOVA analysis of OFT data revealed that splenectomy blocked CRSR-mediated exacerbation of the LPS-induced decrease in time spent in the center (LPS + CRSR: F_1,20_ = 3.161, P = 0.0906; Splenectomy: F_1,20_ = 5.081, P = 0.0356; interaction ((LPS + CRSR) × Splenectomy): F_1,20_ = 4.056, P = 0.0577) and freezing time (LPS + CRSR: F_1,20_ = 6.858, P = 0.0164; Splenectomy: F_1,20_ = 8.777, P = 0.0077; interaction ((LPS + CRSR) × Splenectomy): F_1,20_ = 4.059, P = 0.0576) (Figure 4D, 4E).

Furthermore, splenectomy blocked CRSR-mediated exacerbation of the LPS-induced increase in plasma levels of TNF-α (LPS + CRSR: F_1,20_ = 7.028, P = 0.0153; Splenectomy: F_1,20_ = 5.910, P = 0.0246; interaction ((LPS + CRSR) × Splenectomy): F_1,20_ = 3.633, P = 0.0711), IL-6 (LPS + CRSR: F_1,20_ = 4.542, P = 0.0457; Splenectomy: F_1,20_ = 3.695, P = 0.0689; interaction ((LPS + CRSR) × Splenectomy): F_1,20_ = 3.316, P = 0.0836), IL-17A (LPS + CRSR: F_1,20_ = 6.548, P = 0.0187; Splenectomy: F_1,20_ = 3.810, P = 0.0651; interaction ((LPS + CRSR) × Splenectomy): F_1,20_ = 3.520, P = 0.0753), and IFN-γ (LPS + CRSR: F_1,20_ = 11.70, P = 0.0027; Splenectomy: F_1,20_ = 3.097, P = 0.0937; interaction ((LPS + CRSR) × Splenectomy): F_1,20_ = 3.345, P = 0.0823) (Figure 4F–4I).

### Splenectomy blocked the enhancing effects of CRSR on LPS-induced increase in hippocampal proinflammatory cytokines

**Figure 5:**
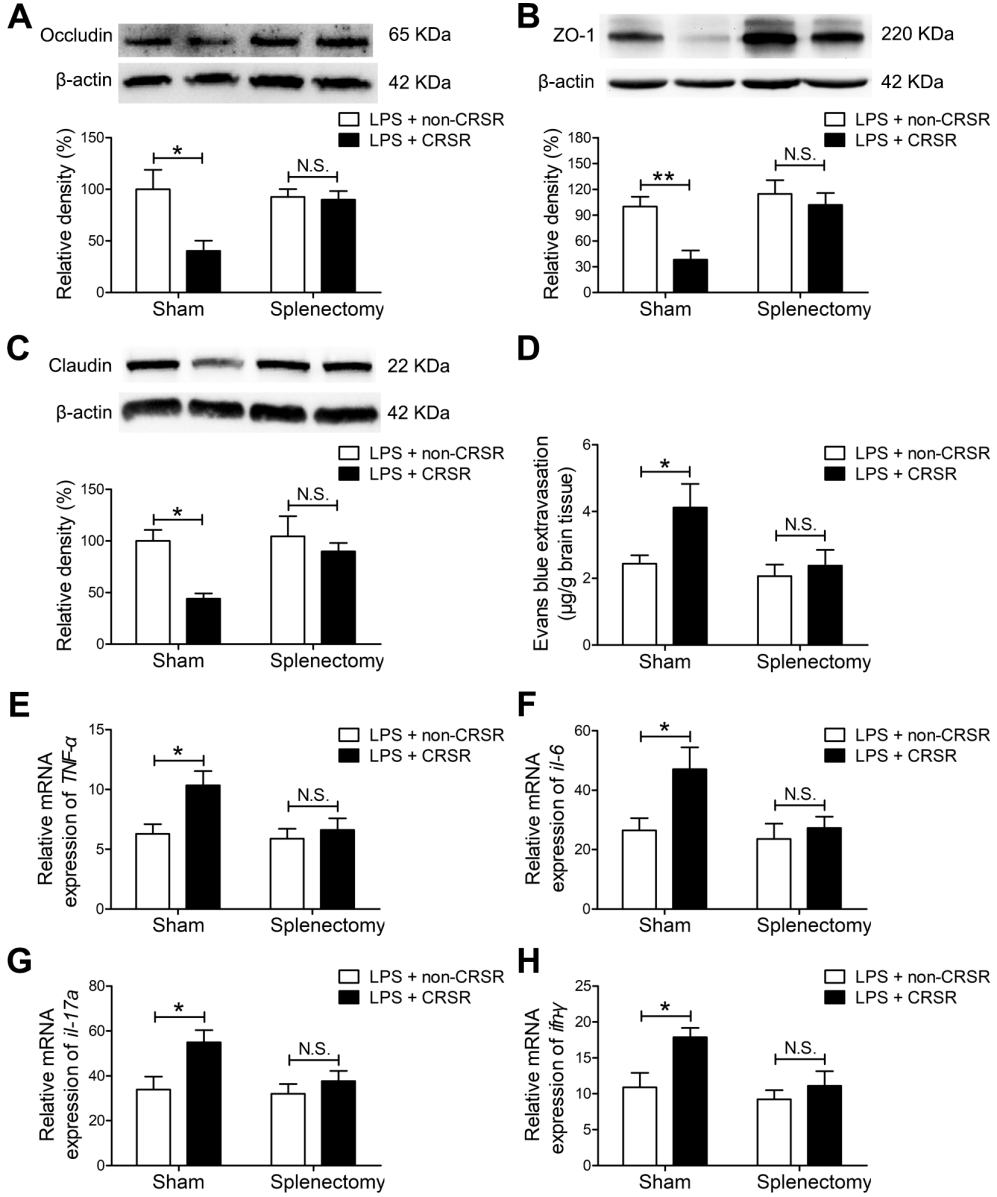
**The spleen mediates the enhancing effects of chronic and repeated short-term sleep restriction (CRSR) on LPS-induced blood-brain barrier (BBB) disruption and hippocampal inflammation 24 hours after LPS treatment**. Western blot analysis of expression of the tight junction proteins occludin (**A**) zona occluden-1 (ZO-1; **B**), and claudin (**C**) in the brain. (**D**) Evans blue dye extravasation test. Splenectomy blocked CRSR-mediated exacerbation of LPS-induced decreases in hippocampal tight junction protein levels and increases in Evans blue dye extravasation into the brain. Quantitative real-time PCR (qRT-PCR) analysis of hippocampal TNF-α (**E**), IL-6 (**F**), IL-17A (**G**), and IFN-γ (**H**) gene expression in each group. Splenectomy blocked CRSR-mediated exacerbation of LPS-induced increases hippocampal expression of these genes. Data represent means ± SEM, n = 6; ^*^P < 0.05, ^**^P < 0.01. N.S., not significant.

Splenectomy blocked CRSR-mediated exacerbation of the LPS-induced decrease in hippocampal expression of occludin (LPS + CRSR: F_1,20_ = 6.698, P = 0.0176; Splenectomy: F_1,20_ = 3.094, P = 0.0939; interaction ((LPS + CRSR) × Splenectomy): F_1,20_ = 5.578, P = 0.0284), ZO-1 (LPS + CRSR: F_1,20_ = 8.499, P = 0.0086; Splenectomy: F_1,20_ = 4.312, P = 0.0509; interaction ((LPS + CRSR) × Splenectomy): F_1,20_ = 2.882, P = 0.1051), and claudin (LPS + CRSR: F_1,20_ = 8.086, P = 0.0100; Splenectomy: F_1,20_ = 8.926, P = 0.0073; interaction ((LPS + CRSR) × Splenectomy): F_1,20_ = 3.477, P = 0.0770) (Figure 5A–5C).

In addition, splenectomy blocked CRSR-mediated exacerbation of the LPS-induced increase in Evans blue extravasation into the brain (LPS + CRSR: F1,20 = 4.353, P = 0.0499; Splenectomy: F1,20 = 4.893, P = 0.0388; interaction ((LPS + CRSR) × Splenectomy: F1,20 = 2.028, P = 0.1698) (Figure 5D).

Splenectomy also blocked CRSR-mediated exacerbation of the LPS-induced increase in mRNA levels of TNF-α (LPS + CRSR: F1,20 = 6.096, P = 0.0227; Splenectomy: F1,20 = 4.564, P = 0.0452; interaction ((LPS + CRSR) × Splenectomy): F1,20 = 2.967, P = 0.1004), IL-6 (LPS + CRSR: F1,20 = 5.268, P = 0.0327; Splenectomy: F1,20 = 4.567, P = 0.0451; interaction ((LPS + CRSR) × Splenectomy): F1,20 = 2.549, P = 0.1260), IL-17A (LPS + CRSR: F1,20 = 6.907, P = 0.0161; Splenectomy: F1,20 = 3.577, P = 0.0732; interaction ((LPS + CRSR) × Splenectomy): F1,20 = 2.318, P = 0.1436), and IFN-γ (LPS + CRSR: F1,20 = 6.734, P = 0.0173; Splenectomy: F1,20 = 6.112, P = 0.0225; interaction ((LPS + CRSR) × Splenectomy): F1,20 = 2.249, P = 0.1494) in the hippocampus (Figure 5E–5H).

### Splenectomy blocked the enhancing effects of CRSR on LPS-induced increase in microglial transition to M1 phenotype

**Figure 6:**
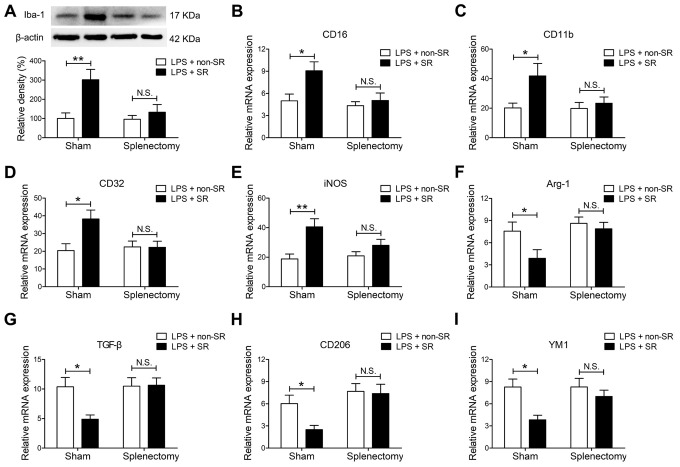
**The spleen mediated the enhancing effects of chronic and repeated short-term sleep restriction (CRSR) on lipopolysaccharide (LPS)-induced transition of microglia to M1 phenotype 24 hours after LPS treatment.** (**A**) Western blot analysis of hippocampal ionized calcium-binding adapter molecule 1 (Iba1) expression in each group. Splenectomy blocked CRSR-mediated exacerbation of the LPS-induced increase in hippocampal Iba1 expression. Quantitative real-time PCR (qRT-PCR) analysis of hippocampal expression of the microglial M1 markers CD16 (**B**), CD11b (**C**), CD32 (**D**), and iNOS (**E**) and the M2 markers Arg-1 (**F**), TGF-β (**G**), CD206 (**H**), and YM1 (**I**). Splenectomy blocked CRSR-mediated exacerbation of LPS-induced increases in microglial M1 markers and decreases in M2 markers in the hippocampus. Data represent means ± SEM, n = 6; ^*^P < 0.05, ^**^P < 0.01. N.S., not significant.

Splenectomy blocked CRSR-mediated exacerbation of the LPS-induced increase in hippocampal Iba1 expression (LPS + CRSR: F_1,20_ = 9.795, P = 0.0053; Splenectomy: F_1,20_ = 5.194, P = 0.0338; interaction ((LPS + CRSR) × Splenectomy): F_1,20_ = 4.720, P = 0.0420) (Figure 6A).

Moreover, splenectomy blocked CRSR-mediated exacerbation of the LPS-induced increase in expression of the microglial M1 markers CD16 (LPS + CRSR: F1,20 = 6.228, P = 0.0214; Splenectomy: F_1,20_ = 5.961, P = 0.0240; interaction ((LPS + CRSR) × Splenectomy): F_1,20_ = 3.124, P = 0.0924), CD11b (LPS + CRSR: F_1,20_ = 5.441, P = 0.0302; Splenectomy: F_1,20_ = 3.077, P = 0.0947; interaction ((LPS + CRSR) × Splenectomy): F_1,20_ = 2.584, P = 0.1067), CD32 (LPS + CRSR: F_1,20_ = 4.907, P = 0.0385; Splenectomy: F_1,20_ = 3.070, P = 0.0951; interaction ((LPS + CRSR) × Splenectomy): F_1,20_ = 5.198, P = 0.0337), and iNOS (LPS + CRSR: F_1,20_ = 12.61, P = 0.0020; Splenectomy: F_1,20_ = 1.661, P = 0.2122; interaction ((LPS + CRSR) × Splenectomy): F_1,20_ = 3.258, P = 0.0861); splenectomy also blocked the CRSR-enhanced LPS-induced decrease in expression of the microglial M2 markers Arg-1 (LPS + CRSR: F_1,20_ = 4.500, P = 0.0466; Splenectomy: F_1,20_ = 5.908, P = 0.0246; interaction ((LPS + CRSR) × Splenectomy): F_1,20_ = 1.983, P = 0.1745), TGF-β (LPS + CRSR: F_1,20_ = 4.362, P = 0.0497; Splenectomy: F_1,20_ = 5.337, P = 0.0317; interaction ((LPS + CRSR) × Splenectomy): F_1,20_ = 4.916, P = 0.0384), CD206 (LPS + CRSR: F_1,20_ = 3.384, P = 0.0807; Splenectomy: F_1,20_ = 9.963, P = 0.0050; interaction ((LPS + CRSR) × Splenectomy): F_1,20_ = 2.429, P = 0.1348), and YM1 (LPS + CRSR: F_1,20_ = 8.944, P = 0.0072; Splenectomy: F_1,20_ = 2.728, P = 0.1142; interaction ((LPS + CRSR) × Splenectomy): F_1,20_ = 2.677, P = 0.1175) (Figure 6B–6I).

## DISCUSSION

**Figure 7:**
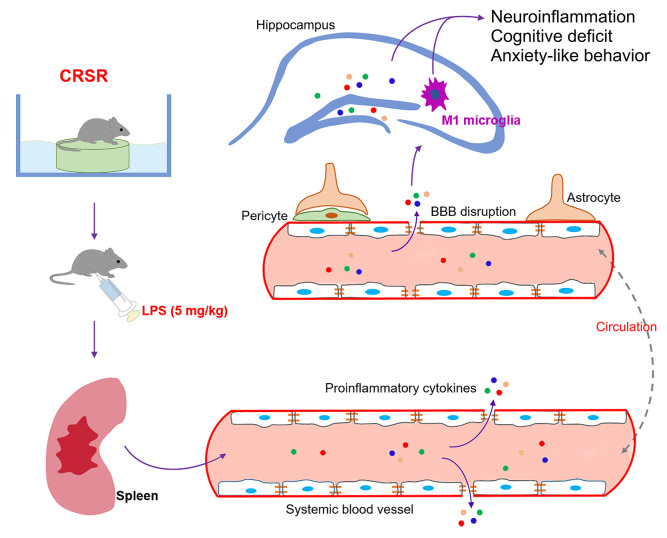
**Schematic illustrating the crucial role of the spleen in exacerbating lipopolysaccharide (LPS)-induced increases in systemic inflammation, neuroinflammation, cognitive deficits, and anxiety-like behavior in mice exposed to CRSR.** BBB, blood-brain barrier; CRSR, chronic and repeated short-term sleep restriction.

The findings of this study indicate that chronic and repeated short-term sleep restriction could exacerbate LPS-induced systemic inflammation, increases in BBB permeability and hippocampal proinflammatory cytokine levels, transition of microglia to the M1 phenotype, cognitive deficits, and anxiety-like behavior. We also identified a novel and critical role for the spleen in the exacerbation of LPS-induced central nervous system damage after CRSR. Interestingly, splenectomy blocked CRSR-mediated exacerbation of LPS-induced increases in hippocampal neuroinflammation, microglial activation and M1 polarization, cognitive deficits, and anxiety-like behavior (Figure 7).

Accumulating evidence indicates that acute short-term or chronic long-term sleep disruption, which are widespread in modern society, can cause systemic inflammation, cellular and humoral immune system dysfunction, and neuroinflammatory responses [12, 15, 24, 32], which collectively can promote synapse loss, mood disorders, cognitive impairment, and neurodegenerative and neurobehavioral diseases [12–15]. Short-term sleep restriction for 3 days increases TNF-α, IL-6, and IL-1β levels in hippocampus and basal forebrain [32]. Chronic sleep deprivation for 21 consecutive days also markedly increased TNF-α and IL-6 expression in hippocampus [15]. However, IL-1β and IL-6 protein expression were not elevated in the hippocampus three weeks after three months of repeated intermittent paradoxical sleep deprivation [24]. Similarly, we found in the present study that plasma and hippocampal levels of TNF-α, IL-6, IL-17A, and IFN-γ were not increased 2 weeks after CRSR compared to non-CRSR control mice, indicating that the enhancing effects of CRSR on LPS-induced neuroinflammatory damage were not associated with pre-existing inflammatory response status. Furthermore, we found that the spleen played essential role in the enhancing effects of CRSR on LPS-induced increases in systemic inflammation and neuroinflammation. Splenectomy two weeks prior to CRSR blocked the enhancing effects of CRSR on LPS-induced neuroinflammatory damage. We speculate that the CRSR-exposed spleen may act as a reservoir of proinflammatory myeloid cells that are released into the blood and brain following LPS treatment, leading to increases in BBB permeability, hippocampal proinflammatory cytokine levels, transition of microglia to the M1 phenotype, cognitive deficits, and anxiety-like behavior. A previous study found that spleens may serve as unique reservoirs of primed monocytes for up to 24 days after six days of repeated social defeat, and accumulated splenic monocytes can be readily released into the blood and travel to the brain soon after acute stress injury [21]. Another study demonstrated that accumulated myeloid cells can migrate from the spleen to tumor tissues after chronic psychological stress, and splenectomy 14 days prior to chronic psychological stress could prevent this cell migration [22]. It is therefore possible that long-term storage of proinflammatory myeloid cells in the spleen after CRSR and trafficking of these cells to brain after LPS stimulation might increase LPS-induced neuroinflammatory damage. Additional studies are needed to confirm whether the spleen enables CRSR-mediated exaggeration of LPS-induced neuroinflammatory injury by storing and releasing proinflammatory myeloid cells.

Systemic inflammation induces cognitive deficits by increasing neuroinflammation, disrupting the BBB, and activating microglia, which are key cellular mediators of neuroinflammatory processes [33–35]. Microglia activated by severe systemic inflammation can trigger either pro-inflammatory (M1) or anti-inflammatory (M2) responses [30]. M1-polarized microglia can promote neuroinflammation by secreting proinflammatory factors like CD16, CD11b, iNOS, and CD32, while M2-polarized microglia can reduce neuroinflammation be releasing anti-inflammatory factors like Arg-1, TGF-β, CD206, and YM1 [36]. Inhibiting M1-polarization and stimulating M2-polarization of microglia exerts protective effects on neuroinflammation and cognitive function [37, 38]. In addition, microglia are both a source and target of cytokines in the central nervous system [39]. In the present study, we found more M1-polarized microglia in the hippocampus of CRSR mice compared to non-CRSR mice after LPS administration, indicating that interactions between M1-polarized microglia and proinflammatory cytokines may contribute to the enhancing effects of CRSR on LPS-induced cognitive deficits and anxiety-like behavior. Furthermore, removal of the spleen blocked CRSR-mediated exacerbation of LPS-induced transition of microglia to M1 phenotype, indicating that the spleen plays a key role in microglial activation and M1 polarization in CRSR-exposed mice.

In summary, we demonstrated for the first time in this study that CRSR increased vulnerability to LPS-induced systemic inflammation, hippocampal neuroinflam-mation, BBB disruption, transition of microglia to the M1 phenotype, cognitive deficits, and anxiety-like behavior in mice. We also identified a unique and essential role for the spleen in the enhancing effects of CRSR on LPS-induced central nervous system damage.

## MATERIALS AND METHODS

### Mice

Seventy-eight week-old male C57BL/6J mice were purchased from Vital River Laboratory Animal Technology Co Ltd., Beijing, China. This age in mice corresponds to about 65 years old in humans [40]. All mice were allowed to acclimate to lab housing for at least 7 days before experiments began. Mice were housed and maintained on a 12 h light-dark diurnal cycle at 22 ± 1 °C. Food and water were provided ad libitum. All procedures were performed in accordance with the National Institute of Health (NIH) Guide for the Care and Use of Laboratory Animals (publications no. 80-23, revised 1996). All animal protocols were approved by the experimental animal committee of Tongji Medical College (permission number: #2018/S255), and all efforts were made to minimize suffering.

### Experimental groups

The mice were randomly assigned to eight groups (n = 6/group). Groups 1-4 (Figure 1A) consisted of LPS or saline-treated mice with or without CRSR (Saline + non-CRSR; LPS + non-CRSR; Saline + CRSR; LPS + CRSR). After mice were subjected to 3 repeated cycles of 7-day sleep restriction with an interval of 7 days, mice exposed to CRSR were allowed to sleep only during the final 4 h of the light phase in every 24 h period using the multiple platform method. Mice were placed in polypropylene cages (500 × 300 × 170 mm; 3 mice/cage) containing 9 circular platforms (diameter 3 cm, height 2.5 cm). The cages were filled with water to 1 cm below the upper surface of the platforms, enabling the mice to move between the platforms and to access food and water during CRSR protocols. At the onset of muscle atonia when mice reached the paradoxical phase of sleep, they fell into the water and were awoken. The cages were cleaned and the water was changed daily. LPS (5 mg/kg; L-4130, serotype 0111:B4; Sigma-Aldrich, St Louis, MO) or 0.9% saline (5 ml/kg) was administrated intraperitoneally 14 days after the last cycle of sleep restriction; this LPS dose was chosen based on a previous study [18]. Experimental groups 5-8 (Figure 4A) consisted of LPS-treated mice subjected to either sham-splenectomy or splenectomy with or without CRSR (Sham + non-CRSR + LPS; splenectomy + non-CRSR + LPS; Sham + CRSR + LPS; splenectomy + CRSR + LPS). Splenectomy surgery was performed 14 days prior to the first cycle of sleep restriction.

### Splenectomy surgery

Total splenectomy was performed under isoflurane anesthesia. A subcostal minimal incision was made on the left dorsolateral side of the abdomen. The afferent and efferent vessels near the spleen were carefully ligated using 6-0 silk suture and the spleen was removed. The abdominal wall was closed with 4-0 silk suture and then the skin was sutured using 3-0 silk suture. Sham-splenectomy mice underwent the same procedure without removal of the spleen. All surgical procedures were performed under sterile conditions. After the surgery, the mice were allowed to recover for two weeks prior to CRSR.

### Functional assays

The open field test (OFT) and Y maze test (YMT) were performed as described in a previous study [41].

Exploratory activity, non-associative memory, and anxiety-like behavior were assessed in the OFT. A video camera was placed directly above the open field chamber (40 × 40 × 40 cm). The mouse was gently placed in the center of the chamber under dim light and was allowed to move freely for 5 minutes. Time (seconds) spent in the center of the field (middle 20 × 20 cm area) and freezing time (seconds) were analyzed. The chamber floor was cleaned with 70% ethanol between each test.

Short-term spatial working memory was assessed using a gray plastic Y maze apparatus consisting of 3 arms (8 cm (width) × 30 cm (length) × 15 cm (height)) with an angle of 120°. The 3 arms included the starting arm, second arm, and novel arm, which was blocked during the first trial and open during the second trial. Different visual cues (circles, triangles, and pentagrams) were placed at the end of each arm and were constant throughout the trials. A video camera was placed directly above the Y maze apparatus. The Y maze test consisted of 2 trials separated by a 2-hour interval. In the first training trial, mouse was allowed to explore the starting arm and second arm for 10 minutes. In the second trial, the mouse was gently placed in the same starting arm and allowed to explore all three arms freely for 5 minutes. The number of entries and time spent in the novel arms were recorded. An arm entry was defined by all four paws of the mouse being within that arm. Each arm was cleaned with 70% ethanol between each trial.

### Evans blue dye extravasation

BBB disruption was evaluated by measuring the extravasation of Evans blue dye as previously described [25]. 24 hours after treatment with LPS or 0.9% saline, mice were injected intraperitoneally with 2% Evans blue dye (5 mL/kg; #E2129, Sigma Chem. Co., St Louis, MO). The dye was then allowed to circulate for 30 min. Mice were then perfused transcardially with phosphate buffered saline (PBS) under deep anesthesia. Whole brains were isolated, weighed, and homogenized in 0.7 mL of PBS. After centrifugation at 15000 × g for 30 minutes, 0.5 mL of the supernatant was collected and added to 0.5 mL of trichloroacetic acid. After overnight incubation at 4 °C and centrifugation at 15000 × g at 4 °C for 30 minutes, the absorbance of the supernatant was measured at 610 nm using a spectrophotometer and was quantified according to a standard curve. The results are presented as micrograms per milligram brain weight.

### ELISA measurement of inflammatory cytokines

Immediately after functional assays, mice were deeply anesthetized with 5% isoflurane, and blood was collected by heart puncture and placed into 1.5 mL ice-cold Eppendorf tubes containing EDTA. Blood samples were immediately centrifuged at 3000 × g for 5 min to prepare plasma samples which were stored at -80 °C until bioanalysis. Plasma levels of tumor necrosis factor alpha (TNF-α; #88-7324, Invitrogen, Camarillo, CA, USA), interleukin (IL)-6 (#88-7064, Invitrogen, Camarillo, CA, USA), IL-17A (#RK00039, ABclonal, Wuhan, China), and interferon-gamma (IFN-γ; #88-8314, Invitrogen, Camarillo, CA, USA) were measured using Enzyme-Linked ImmunoSorbent Assay (ELISA) kits according to the manufacturer’s protocol.

### Western blotting

Immediately after functional assays, bilateral hippocampal tissues were collected and frozen in liquid nitrogen. The hippocampal samples were then lysed in RIPA lysis buffer containing protease inhibitors (KeyGen Biotech, Nanjing, China). Total protein concentrations were measured using the BCA assay (Beyotime Biotechnology, Shanghai, China). Proteins from each sample (50 μg) were separated on 10% SDS-PAGE gels (Beyotime Institute of Biotechnology, Shanghai, China) and transferred to polyvinylidene difluoride membranes (Millipore; Merck KGaA) using an electrophoresis apparatus (Bio-Rad Laboratories, Inc., Hercules, CA, USA). Membranes were blocked in 5% nonfat milk diluted in Tris-buffered saline containing 0.1% Tween-20 (TBST) at room temperature for 1 hour. The membranes were subsequently incubated overnight at 4°C with primary antibodies against occludin (1:1,000, #A12621, ABclonal, Wuhan, China), zona occluden-1 (ZO-1; 1:500, #61-7300, Thermo Fisher Scientific, Waltham, MA), claudin (1:1000, #A11530, ABclonal, Wuhan, China), ionized calcium-binding adapter molecule 1 (Iba1; 1:1000, Wako, Osaka, Japan), and β-actin (1:2000, Santa Cruz Biotechnology, Inc., Dallas, TX, USA). After three washes with TBST, membranes were incubated with horseradish peroxidase-conjugated goat anti-rabbit antibody for 2 hours at room temperature. Chemiluminescent signals were visualized using electrochemiluminescence Western blotting detection reagents (Millipore; Merck KGaA) and bands were captured using a UVP gel documentation system (UVP, LLC, Phoenix, AZ, USA). Band intensity was quantified using Image J software (version 1.41; National Institutes of Health, Bethesda, MD, USA).

### Quantitative real-time PCR (qRT-PCR)

Immediately after functional assays, bilateral hippocampal tissues were collected and placed in liquid nitrogen. Total RNA was extracted from hippocampal samples using Trizol Regent (Invitrogen, Carlsbad, CA). cDNA was then synthesized using a reverse transcription kit (Applied Biosystems, Foster City, CA) and amplified with Power SYBR Green (Applied Biosystems, Foster City, CA). Reactions were run and analyzed on an ABI StepOne™ Real-Time PCR System (Applied Biosystems, Foster City, CA, USA). The thermal cycling program consisted of a single hold at 95 °C for 10 min followed by 40 cycles of 95 °C for 5 s, 55 °C for 30 s, and 72 °C for 60 s. β-actin mRNA levels were used as the internal control. The relative quantitation value is expressed as 2–ΔCt, where ΔCt is the difference between the mean ΔCt value of duplicate measurements of the sample and the β-actin control. All primers used in this study are listed in Table 1.

**Table 1:** qRT-PCR Primers.

**Gene**	**Forward (5’-3’)**	**Reverse (5’-3’)**
CD16	TTTGGACACCCAGATGTTTCAG	GTCTTCCTTGAGCACCTGGATC
CD11b	CCAAGACGATCTCAGCATCA	TTCTGGCTTGCTGAATCCTT
CD32	AATCCTGCCGTTCCTACTGATC	GTGTCACCGTGTCTTCCTTGAG
iNOS	CAAGCACCTTGGAAGAGGAG	AAGGCCAAACACAGCATACC
Arg-1	TCACCTGAGCTTTGATGTCG	CTGAAAGGAGCCCTGTCTTG
TGF-β	GTGTGGAGCAACATGTGGAACTCTA	TTGGTTCAGCCACTGCCGTA
CD206	CAAGGAAGGTTGGCATTTGT	CCTTTCAGTCCTTTGCAAGC
YM1	CAGGGTAATGAGTGGGTTGG	CACGGCACCTCCTAAATTGT
TNF-α	AGAAGTTCCCAAATGGCCTC	TTTTCACAGGGGAGAAATCG
IL-6	GAGGATACCACTCCCAACAGACC	GAGGGATATCTATCAGG GTCTTCAT
IL-17A	TGTGAAGGTCAACCTCAAAGTCT	GAGGGATATCTATCAGG GTCTTCAT
IFN-γ	TCAAGTGGCATAGATGTGGAAGA	GAGATAATCTGGCTCTGCAGGATT
β-actin	AAGGCCAACCGTGAAAAGAT	GTGGTACGACCAGAGGCATAC

### Statistical analysis

Data were analyzed using two-way analysis of variance (ANOVA) followed by Bonferroni's post-hoc test. All data are presented as means ± standard error of the mean (SEM). A P value of less than 0.05 was considered statistically significant. Statistical analyses were performed using SPSS version 20.0 software (SPSS, Tokyo, Japan).

## References

[1] Dang-Vu TT, Desseilles M, Peigneux P, Maquet P. A role for sleep in brain plasticity. Pediatr Rehabil. 2006; 9:98–118. 10.1080/13638490500138702 16449068

[2] Haspel JA, Anafi R, Brown MK, Cermakian N, Depner C, Desplats P, Gelman AE, Haack M, Jelic S, Kim BS, Laposky AD, Lee YC, Mongodin E, et al. Perfect timing: circadian rhythms, sleep, and immunity - an NIH workshop summary. JCI Insight. 2020; 5:e131487. 10.1172/jci.insight.131487 31941836 PMC7030790

[3] McCoy JG, Strecker RE. The cognitive cost of sleep lost. Neurobiol Learn Mem. 2011; 96:564–82. 10.1016/j.nlm.2011.07.004 21875679 PMC3614362

[4] Goel N, Rao H, Durmer JS, Dinges DF. Neurocognitive consequences of sleep deprivation. Semin Neurol. 2009; 29:320–39. 10.1055/s-0029-1237117 19742409 PMC3564638

[5] Stickgold R. Neuroscience: a memory boost while you sleep. Nature. 2006; 444:559–60. 10.1038/nature05309 17086196

[6] Zhang J, Zhu Y, Zhan G, Fenik P, Panossian L, Wang MM, Reid S, Lai D, Davis JG, Baur JA, Veasey S. Extended wakefulness: compromised metabolics in and degeneration of locus ceruleus neurons. J Neurosci. 2014; 34:4418–31. 10.1523/JNEUROSCI.5025-12.2014 24647961 PMC3960479

[7] Walker MP. The role of sleep in cognition and emotion. Ann N Y Acad Sci. 2009; 1156:168–97. 10.1111/j.1749-6632.2009.04416.x 19338508

[8] Walker MP. Cognitive consequences of sleep and sleep loss. Sleep Med. 2008 (Suppl 1); 9:S29–34. 10.1016/S1389-9457(08)70014-5 18929316

[9] Miyamoto M. Pharmacology of ramelteon, a selective MT1/MT2 receptor agonist: a novel therapeutic drug for sleep disorders. CNS Neurosci Ther. 2009; 15:32–51. 10.1111/j.1755-5949.2008.00066.x 19228178 PMC2871175

[10] Mander BA, Winer JR, Walker MP. Sleep and human aging. Neuron. 2017; 94:19–36. 10.1016/j.neuron.2017.02.004 28384471 PMC5810920

[11] Brown SA, Schmitt K, Eckert A. Aging and circadian disruption: causes and effects. Aging (Albany NY). 2011; 3:813–17. 10.18632/aging.100366 21869460 PMC3184982

[12] Besedovsky L, Lange T, Haack M. The sleep-immune crosstalk in health and disease. Physiol Rev. 2019; 99:1325–80. 10.1152/physrev.00010.2018 30920354 PMC6689741

[13] Kincheski GC, Valentim IS, Clarke JR, Cozachenco D, Castelo-Branco MT, Ramos-Lobo AM, Rumjanek VM, Donato J Jr, De Felice FG, Ferreira ST. Chronic sleep restriction promotes brain inflammation and synapse loss, and potentiates memory impairment induced by amyloid-β oligomers in mice. Brain Behav Immun. 2017; 64:140–51. 10.1016/j.bbi.2017.04.007 28412140

[14] Zhu Y, Zhan G, Fenik P, Brandes M, Bell P, Francois N, Shulman K, Veasey S. Chronic sleep disruption advances the temporal progression of tauopathy in P301S mutant mice. J Neurosci. 2018; 38:10255–70. 10.1523/JNEUROSCI.0275-18.2018 30322903 PMC6262148

[15] Manchanda S, Singh H, Kaur T, Kaur G. Low-grade neuroinflammation due to chronic sleep deprivation results in anxiety and learning and memory impairments. Mol Cell Biochem. 2018; 449:63–72. 10.1007/s11010-018-3343-7 29549603

[16] Huang WY, Liu KH, Lin S, Chen TY, Tseng CY, Chen HY, Wu HM, Hsu KS. NADPH oxidase 2 as a potential therapeutic target for protection against cognitive deficits following systemic inflammation in mice. Brain Behav Immun. 2020; 84:242–52. 10.1016/j.bbi.2019.12.006 31841660

[17] Banks WA, Gray AM, Erickson MA, Salameh TS, Damodarasamy M, Sheibani N, Meabon JS, Wing EE, Morofuji Y, Cook DG, Reed MJ. Lipopolysaccharide-induced blood-brain barrier disruption: roles of cyclooxygenase, oxidative stress, neuroinflammation, and elements of the neurovascular unit. J Neuroinflammation. 2015; 12:223. 10.1186/s12974-015-0434-1 26608623 PMC4660627

[18] Sorrenti V, Contarini G, Sut S, Dall’Acqua S, Confortin F, Pagetta A, Giusti P, Zusso M. Curcumin prevents acute neuroinflammation and long-term memory impairment induced by systemic lipopolysaccharide in mice. Front Pharmacol. 2018; 9:183. 10.3389/fphar.2018.00183 29556196 PMC5845393

[19] Ringgold KM, Barf RP, George A, Sutton BC, Opp MR. Prolonged sleep fragmentation of mice exacerbates febrile responses to lipopolysaccharide. J Neurosci Methods. 2013; 219:104–12. 10.1016/j.jneumeth.2013.07.008 23872243 PMC3993011

[20] Wohleb ES, McKim DB, Shea DT, Powell ND, Tarr AJ, Sheridan JF, Godbout JP. Re-establishment of anxiety in stress-sensitized mice is caused by monocyte trafficking from the spleen to the brain. Biol Psychiatry. 2014; 75:970–81. 10.1016/j.biopsych.2013.11.029 24439304 PMC4084643

[21] McKim DB, Patterson JM, Wohleb ES, Jarrett BL, Reader BF, Godbout JP, Sheridan JF. Sympathetic release of splenic monocytes promotes recurring anxiety following repeated social defeat. Biol Psychiatry. 2016; 79:803–13. 10.1016/j.biopsych.2015.07.010 26281717 PMC4728074

[22] Jiang W, Li Y, Li ZZ, Sun J, Li JW, Wei W, Li L, Zhang C, Huang C, Yang SY, Yang J, Kong GY, Li ZF. Chronic restraint stress promotes hepatocellular carcinoma growth by mobilizing splenic myeloid cells through activating β-adrenergic signaling. Brain Behav Immun. 2019; 80:825–38. 10.1016/j.bbi.2019.05.031 31125710

[23] Jiang W, Li Y, Sun J, Li L, Li JW, Zhang C, Huang C, Yang J, Kong GY, Li ZF. Spleen contributes to restraint stress induced changes in blood leukocytes distribution. Sci Rep. 2017; 7:6501. 10.1038/s41598-017-06956-9 28747688 PMC5529540

[24] Yin M, Chen Y, Zheng H, Pu T, Marshall C, Wu T, Xiao M. Assessment of mouse cognitive and anxiety-like behaviors and hippocampal inflammation following a repeated and intermittent paradoxical sleep deprivation procedure. Behav Brain Res. 2017; 321:69–78. 10.1016/j.bbr.2016.12.034 28043900

[25] Jiao H, Wang Z, Liu Y, Wang P, Xue Y. Specific role of tight junction proteins claudin-5, occludin, and ZO-1 of the blood-brain barrier in a focal cerebral ischemic insult. J Mol Neurosci. 2011; 44:130–39. 10.1007/s12031-011-9496-4 21318404

[26] Nishikawa H, Liu L, Nakano F, Kawakita F, Kanamaru H, Nakatsuka Y, Okada T, Suzuki H. Modified Citrus Pectin Prevents Blood-Brain Barrier Disruption in Mouse Subarachnoid Hemorrhage by Inhibiting Galectin-3. Stroke. 2018; 49:2743–2751. 10.1161/STROKEAHA.118.021757 30355205

[27] Saunders NR, Dziegielewska KM, Møllgård K, Habgood MD. Markers for blood-brain barrier integrity: how appropriate is evans blue in the twenty-first century and what are the alternatives? Front Neurosci. 2015; 9:385. 10.3389/fnins.2015.00385 26578854 PMC4624851

[28] Banks WA. The blood-brain barrier in neuroimmunology: tales of separation and assimilation. Brain Behav Immun. 2015; 44:1–8. 10.1016/j.bbi.2014.08.007 25172555 PMC4275374

[29] Opp MR, George A, Ringgold KM, Hansen KM, Bullock KM, Banks WA. Sleep fragmentation and sepsis differentially impact blood-brain barrier integrity and transport of tumor necrosis factor-α in aging. Brain Behav Immun. 2015; 50:259–65. 10.1016/j.bbi.2015.07.023 26218294 PMC4831867

[30] Michels M, Abatti MR, Ávila P, Vieira A, Borges H, Carvalho Junior C, Wendhausen D, Gasparotto J, Tiefensee Ribeiro C, Moreira JC, Gelain DP, Dal-Pizzol F. Characterization and modulation of microglial phenotypes in an animal model of severe sepsis. J Cell Mol Med. 2020; 24:88–97. 10.1111/jcmm.14606 31654493 PMC6933367

[31] He J, Crews FT. Increased MCP-1 and microglia in various regions of the human alcoholic brain. Exp Neurol. 2008; 210:349–58. 10.1016/j.expneurol.2007.11.017 18190912 PMC2346541

[32] Zielinski MR, Kim Y, Karpova SA, McCarley RW, Strecker RE, Gerashchenko D. Chronic sleep restriction elevates brain interleukin-1 beta and tumor necrosis factor-alpha and attenuates brain-derived neurotrophic factor expression. Neurosci Lett. 2014; 580:27–31. 10.1016/j.neulet.2014.07.043 25093703 PMC4162816

[33] Streit WJ, Mrak RE, Griffin WS. Microglia and neuroinflammation: a pathological perspective. J Neuroinflammation. 2004; 1:14. 10.1186/1742-2094-1-14 15285801 PMC509427

[34] Cianciulli A, Porro C, Calvello R, Trotta T, Lofrumento DD, Panaro MA. Microglia mediated neuroinflammation: focus on PI3K modulation. Biomolecules. 2020; 10:137. 10.3390/biom10010137 31947676 PMC7022557

[35] Michels M, Ávila P, Pescador B, Vieira A, Abatti M, Cucker L, Borges H, Goulart AI, Junior CC, Barichello T, Quevedo J, Dal-Pizzol F. Microglial cells depletion increases inflammation and modifies microglial phenotypes in an animal model of severe sepsis. Mol Neurobiol. 2019; 56:7296–304. 10.1007/s12035-019-1606-2 31020614

[36] Tang Y, Le W. Differential roles of M1 and M2 microglia in neurodegenerative diseases. Mol Neurobiol. 2016; 53:1181–94. 10.1007/s12035-014-9070-5 25598354

[37] Zhuang X, Yu Y, Jiang Y, Zhao S, Wang Y, Su L, Xie K, Yu Y, Lu Y, Lv G. Molecular hydrogen attenuates sepsis-induced neuroinflammation through regulation of microglia polarization through an mTOR-autophagy-dependent pathway. Int Immunopharmacol. 2020; 81:106287. 10.1016/j.intimp.2020.106287 32058932

[38] Tian M, Qingzhen L, Zhiyang Y, Chunlong C, Jiao D, Zhang L, Li W. Attractylone attenuates sepsis-associated encephalopathy and cognitive dysfunction by inhibiting microglial activation and neuroinflammation. J Cell Biochem. 2019. [Epub ahead of print]. 10.1002/jcb.27983 30672013

[39] Hanisch UK. Microglia as a source and target of cytokines. Glia. 2002; 40:140–55. 10.1002/glia.10161 12379902

[40] Sharon G, Sampson TR, Geschwind DH, Mazmanian SK. The central nervous system and the gut microbiome. Cell. 2016; 167:915–32. 10.1016/j.cell.2016.10.027 27814521 PMC5127403

[41] Peng M, Zhang C, Dong Y, Zhang Y, Nakazawa H, Kaneki M, Zheng H, Shen Y, Marcantonio ER, Xie Z. Battery of behavioral tests in mice to study postoperative delirium. Sci Rep. 2016; 6:29874. 10.1038/srep29874 27435513 PMC4951688

